# Soil-Derived Microbial Consortia Enriched with Different Plant Biomass Reveal Distinct Players Acting in Lignocellulose Degradation

**DOI:** 10.1007/s00248-015-0683-7

**Published:** 2015-10-20

**Authors:** Maria Julia de Lima Brossi, Diego Javier Jiménez, Larisa Cortes-Tolalpa, Jan Dirk van Elsas

**Affiliations:** Department of Microbial Ecology, Groningen Institute for Evolutionary Life Sciences, University of Groningen, Nijenborgh 7, 9747AG Groningen, The Netherlands

**Keywords:** Plant biomass, Bioconversion, Bacterial–fungal consortia, (Hemi) cellulolytic activity

## Abstract

Here, we investigated how different plant biomass, and—for one substrate—pH, drive the composition of degrader microbial consortia. We bred such consortia from forest soil, incubated along nine aerobic sequential - batch enrichments with wheat straw (WS1, pH 7.2; WS2, pH 9.0), switchgrass (SG, pH 7.2), and corn stover (CS, pH 7.2) as carbon sources. Lignocellulosic compounds (lignin, cellulose and xylan) were best degraded in treatment SG, followed by CS, WS1 and WS2. In terms of composition, the consortia became relatively stable after transfers 4 to 6, as evidenced by PCR-DGGE profiles obtained from each consortium DNA. The final consortia differed by ~40 % (bacteria) and ~60 % (fungi) across treatments. A ‘core’ community represented by 5/16 (bacteria) and 3/14 (fungi) bands was discerned, next to a variable part. The composition of the final microbial consortia was strongly driven by the substrate, as taxonomically-diverse consortia appeared in the different substrate treatments, but not in the (WS) different pH one. Biodegradative strains affiliated to *Sphingobacterium kitahiroshimense*, *Raoultella terrigena*, *Pseudomonas putida*, *Stenotrophomonas rhizophila* (bacteria), *Coniochaeta ligniaria* and *Acremonium* sp. (fungi) were recovered in at least three treatments, whereas strains affiliated to *Delftia tsuruhatensis*, *Paenibacillus xylanexedens*, *Sanguibacter inulus* and *Comamonas jiangduensis* were treatment-specific.

**Electronic supplementary material:**

The online version of this article (doi:10.1007/s00248-015-0683-7) contains supplementary material, which is available to authorized users.

## Introduction

Wheat straw (WS), corn stover (CS), and switchgrass (SG) constitute excellent sources of lignocellulose with high potential for the production of useful compounds such as biofuel, polyolefin-based plastics and lactic acid. Lignocellulose is mainly composed of cellulose, hemicellulose, lignin and pectin [10, 29]. Its composition in plant matter can vary according to plant type, and even within plant species [2], which affects its bioconversion [14, 15]. Thus, one may surmise that WS, CS, and SG substrates potentially require diverse specialized combinations of microorganisms for its deconstruction [6, 30, 37, 39]. It is currently accepted that proper biodegradation of lignocellulosic substrates requires a complex set of enzymes. Thus, peroxidases, laccases, endoglucanases, exoglucanases, β-glucosidases, fucosidases and xylanases [28, 40, 42], among other enzymes, may be required in different and fluctuating amounts and proportions. Moreover, cultures from pure isolates have often demonstrated unsatisfactory biodegradation rates [20, 21]. Hence, recent work has focused on plant biomass degradation by microbial consortia on the premise that the expected diversity of the microbially - secreted enzymes will result in efficient degradation rates [34]. The microbial groups involved may even be interdependent, with each one exerting distinct functions, the sum of which is synergistic for the process [23]. And, as a result, the microbial consortia may also better withstand physiological fluctuations. Examining the microbial consortia bred on lignocellulosic plant biomass is useful for (1) understanding (2) designing, and (3) testing superior biodegradative agents [43].

To produce such superior microbial consortia, the dilution-to-stimulation approach, which uses sequential - batch enrichments on the same substrate, is indicated [16] as it allows to establish stable microbial consortia with desirable biodegradation properties [5, 18, 27]. Effective consortia can be readily derived from a source community from forest soil [8, 18]. However, in the light of the richness of lignocellulose biodegradative capacities in forest soil, it is important to assess to what extent the choice of lignocellulosic substrates (e.g., WS, CS, or SG) directs the assembly of efficient degrading microbial consortia.

Here, we hypothesized that, given the overall similarity in substrate composition, microbial consortia with largely similar structures will be produced from one source community in a sequential batch dilution-to-stimulation approach. However, an alternative hypothesis postulates that such communities are bound to be different in the light of the—possibly subtle—differences in substrate composition. Following these two divergent lines of reasoning, the main objectives of this study were (1) to produce effective microbial consortia on the aforementioned three substrates and (2) to test whether these diverse substrates (next to variation in pH for the WS treatment) drive the establishment of different (or similar) microbial lignocellulose-degrading consortia.

## Materials and Methods

### Plant Biomass Preparation

We collected approximately 3 kg of each plant biomass—i.e., wheat straw, switchgrass and corn stover—in local farms in Groningen, The Netherlands. Each plant biomass raw material was transported to the laboratory (<24 h) at room temperature (20 °C) for further processing. The raw material was air-dried at 50 °C for 24 h before grinding using a hammer mill, yielding pieces <1 mm.

The experimental design encompassed three different treatments with respect to the plant biomass used (a proxy for different carbon sources), next to one (with WS), in which pH was varied as follows: wheat straw (WS1), switchgrass (SG), and corn stover (CS)—all maintained at pH 7.2, and wheat straw (WS2) under a pH 9.0. All treatments were performed in triplicate flasks (*n* = 3), and all flasks were kept under the same conditions along the whole experiment to avoid bias.

### Serial Batch Enrichment Cultures Using the Dilution-to-Stimulation Approach

Ten randomly taken soil samples of 10 g were collected from a forest soil (0 to 10 cm depth) in Groningen, the Netherlands (53.41 N; 6.90 E) in September, 2013 (before leaf abscission). These samples were mixed to produce one representative soil sample to be used as the source inoculum for all treatments. The soil sample was transported to the laboratory at room temperature (20 °C) for further processing (<24 h). Cell suspensions were prepared by adding 10 g of the mixed soil to an Erlenmeyer flask (250 mL) containing 10 g of sterile gravel in 90 mL of 0.9 % saline (NaCl). The flask was shaken for 20 min at 250 rpm, and 3 mL of cell suspension was then sampled and frozen (−20 °C) for total DNA extraction. Moreover, aliquots (150 μL) of the cell suspension were added to triplicate flasks containing 15 mL of mineral salt medium (MSM), pH 7.2 for treatments WS1, SG, and CS and pH 9.0 for treatment WS2, with 1 % of the respective lignocellulosic substrates; all flasks were supplemented with 15 μL of standard trace element and vitamin solution. For a detailed description of this method, see Jiménez et al. [18]. Subsequently, flasks were incubated at 28 °C with shaking at 150 rpm. Two controls, i.e., one without substrate and one without microbial source (for all substrates) were also set up. Cultures were monitored for growth at regular times, and once the systems reached high cell density (10^7^–10^8^ cells mL^−1^) (between 5 and 6 days), aliquots (15 μL) were transferred to 15 mL of fresh medium (lignocellulose source in MSM supplemented with vitamins and trace elements) thus giving a dilution of 10^−3^. This procedure was repeated nine times, giving in total nine enrichments. Cell counts were obtained by microscopy using a *Bürke-Turk* chamber (Blaubrand^®^) according to a standard protocol. The quantification was done directly after the transfer and at the end of growth in each transfer. The pH values of all treatments were regularly monitored and revealed to be largely stable along the incubation period.

Finally, samples were taken from the consortia, at the end of growth in each transfer, being one 2-mL aliquot (from each flask—*n* = 3) for DNA extraction and another 1-mL aliquot stored in glycerol (25 %) at −80 °C.

### Total DNA Extraction and Quantitative PCR

DNA extractions from the consortia were performed using 2 mL of each sample. The UltraClean Microbial DNA Isolation kit (MoBio^®^ Laboratories Inc., Carlsbad, USA) was used according to the manufacturer’s instructions. Bacterial and fungal counts were obtained by quantifying, respectively, the bacterial 16S rRNA gene (regions V5–V6) and the fungal first internal transcribed spacer (ITS1) region by quantitative PCR (qPCR) using 5 ng of extracted consortial DNA as the template and the primer sets 16SFP/16SRP and 5.8S/ITS1 [31]. Standard curves were constructed using serial dilutions of plasmids (1 to 8 log copies μL^−1^) that contained cloned bacterial 16S rRNA gene and ITS1 fragments from *Serratia plymuthica* (KF495530) and *Coniochaeta ligniaria* (KF285995), respectively. Absolute quantification was carried out in three replicates on an ABI Prism 7300 Cycler (Applied Biosystem, Lohne, Germany). The bacterial and fungal abundances in the different samples were expressed as target gene copy numbers per milliliter. Statistical comparisons between the means were performed using one-way ANOVA (Tukey’s test).

### Substrate Weight Loss

At the end of transfers 6 and 9, the residual solid matter in the cultures was washed and dried as described in Du et al. [11], after which the weight of the residual matter was measured and compared to a reference control treatment without the inoculum. The percentage of weight loss was defined as the ratio of the weight loss compared to the initial weight (%) as calculated by the following formula:

Substrate weight loss (%) = [(*a* − *b*) / *c*] × 100; where: *a* = residual control substrate weight; *b* = residual substrate weight; *c* = total substrate weight.

Statistical comparisons of the samples’ substrate weight losses were performed using one-way ANOVA of the means per treatment (Tukey’s test).

### Lignocellulosic Composition of Substrates and Degradation Rate

In order to determine the composition of each substrate and the degradation rate of their lignocellulosic components, we used fourier transformed infrared (FTIR) spectroscopy [1]. To do so, for all used substrates (i.e., WS, SG, and CS), we quantified the percentages of lignin, cellulose and hemicellulose (i.e., xylan from birchwood as the proxy) content before and after incubation (transfer nine).

Prior to quantification, the material from the triplicates of each treatment (WS1, WS2, SG, and CS) was individually dried at 50 °C for 24 h. Standard curves were determined using mixed components (i.e., lignin, cellulose and xylan) in eight different proportions (Table S1); this resulted in reference spectra and validation of the prediction of the lignocellulosic components. The compounds were measured using an FTIR spectrum machine (Thermo Fisher Scientific). The data were preprocessed using Savitzky–Golay differentiation (second derivative; polynomial order 2 and 31-point curve employed for each correction) in order to fit a polynomial regression to each successive curve segment. This generated smoothed curves [33], followed a standard normal variate (SNV) to transform centers and scales of each individual spectrum [9]. After preprocessing, spectrum analyses were conducted, creating a partial least squares regression model using the standard curve, including an FTIR wavelength from 800 to 1800 cm^−1^ [12, 22]. The predictive model displayed *R*^2^ values of 0.95, 0.96 and 0.97 for lignin, cellulose and hemicellulose, respectively. All quantitative values are expressed in percentages of each compound (lignin, hemicellulose, and cellulose) presented in each substrate. Data analyses were performed using the “Unscrambler” software (CAMO Software, 2011). Finally, degradation rates were determined, expressed as the ratio of the percentage of each component in the substrate after incubation compared to that before incubation as follows:

Degradation rate (%) = [(*a* − *b*) / *a*] × 100; where *a* = percentage of component in the substrate before incubation and *b* = percentage of component in the substrate after incubation.

Statistical comparisons of the mean’ degradation rates were performed using one-way ANOVA (Tukey’s test).

### PCR-DGGE analysis

Bacterial and fungal community structures were assessed by PCR-denaturing gradient gel electrophoresis (PCR-DGGE) of the total consortium DNA along transfers 1, 4, 6 and 9 (T1, T4, T6, and T9) in all treatments. Thus, PCR-DGGE enabled the evaluation of consortial development and stability during the enrichment process as well as the identification shifts among the final consortium profiles. The microbial consortia were considered to be stable when the community structures (for bacteria or fungi) presented a similar pattern along at least three sequential transfers. In order to provide taxonomic information of specific bands found in our DGGE patterns, we performed a co-migration analysis. Briefly, 16S rRNA gene sequences were amplified for key selected consortium strains (see later) using DGGE primers, after which the resulting amplicons were run in parallel with the consortium amplicons. Bands that co-migrated with consortium bands were considered to presumptively identify organisms in the consortium patterns.

DGGE was performed in the Ingeny Phor-U System (Ingeny International, Goes, The Netherlands). PCR was performed with primers F968-GC clamp and R1401.1b for the bacterial 16S rRNA gene. For fungal communities, primers EF4/ITS4 were used in the first PCR, which was followed by a second amplification with the primers ITS1f-GC/ITS2. Primer sequences, PCR mixtures and cycling conditions were used as previously described [31]. The DGGE was performed in 6 % (*w*/*v*) polyacrylamide gels with 45–65 and 20–50 % denaturant gradients for bacterial and fungal communities, respectively (100 % is defined as 7 M urea with 40 % deionized formamide). Electrophoresis was carried out at 100 V for 16 h at 60 °C, and the gels were stained for 30 min in 0.5 % TAE buffer with SYBR gold (final concentration of 0.5 μg L^−1^) (Invitrogen, Breda, The Netherlands). Images were taken using Imagemaster VDS (Amersham Biosciences, Buckinghamshire, UK). Fingerprinting results were analyzed using the GelCompar software (Applied Maths, Sint- Martens Latem, Belgium). The quantity of bands for each treatment was considered as a proxy roughly reporting on phylotype richness. We avoided quantifying band intensities since it may introduce bias into the analyses according to differences obtained in DNA templates and/or PCR efficiencies. Thus, presence/absence of band patterns were converted to Jaccard dissimilarity matrices for non-metric multidimensional scaling (nMDS) followed by the analysis of similarities (ANOSIM) statistical analysis using Primer6 (PrimerE, Ivybridge, UK). The global *R* values, generated by ANOSIM, can range from −1 to 1; objects that are more dissimilar between groups than within groups are indicated by an *R* greater than 0; an *R* value of 0 indicates that the null hypothesis of no difference is true [13].

### Isolation and Identification of Bacterial and Fungal Strains

Bacterial and fungal isolates were obtained from transfer 9 of all treatments on R2A agar (BD Difco^®^, Detroit, USA) and potato dextrose agar (PDA) (Duchefa Biochemie BV, Haarlem, the Netherlands), respectively. Serial dilutions were performed in MSM, and 100 μL of dilutions 10^−5^ to 10^−8^ were spread on the surface of each medium. Bacterial and fungal colonies with different morphologies were subsequently subcultured (aerobically) to purity. Totals of 11, 8, 9 and 8 bacterial and 4, 3, 3 and 3 fungal strains were thus isolated from treatments WS1, WS2, SC and CS, respectively. Bacterial isolates were preserved at 4 °C (on solid R2A medium) and −80 °C (liquid R2A medium in glycerol 25 %), while fungal ones were cut from the solid medium (25 mm^2^ squares), after which they were preserved in distilled water at room temperature (Castellani method). The UltraClean Microbial DNA Isolation kit (MoBio^®^ Laboratories Inc., Carlsbad, USA) was used according to the manufacturer’s instructions for genomic DNA extractions. The bacterial 16S rRNA genes were PCR amplified using 5 ng of DNA and primers B8F and 1406R according to Taketani et al. [38]. For fungal strains, we amplified the partial 18S rRNA gene using primers EF4 and ITS4 according to Jiménez et al. [18]. PCR products were sequenced by Sanger technology (LGC Genomics, Germany) using the 1406R primer (for bacteria) and ITS4 primer (for fungi). All resulting chromatograms were analyzed for quality using the Lucy algorithm (RDP website; http://rdp.cme.msu.edu/). In this, quality trimming by removing bases with low scores was applied. The level of minimum requirement was 400 bp with quality above 20 (phred score—one error per 100 bases read). Taxonomic assignment of the sequences was done using BLAST-N against the National Center for Biotechnology Information (NCBI) database (http://blast.ncbi.nlm.nih.gov/Blast.cgi). Sequences are publicly available in the GenBank database under accession numbers KR935800 to KR935847.

### Screening of Strains for (Hemi)cellulolytic Activity

Screenings for (hemi)cellulolytic activity were done in mineral medium agar (MMA) (0.2 % NaNO_3_; 0.1 % K_2_HPO_4_; 0.05 % MgSO_4_; 0.05 % KCl; 1 % of vitamin solution; 1.5 % agar). We evaluated the growth (negative, weak and positive) of the strains in the presence of 0.2 % glucose (positive control), 0.2 % carboxymethyl cellulose (CMC; Sigma-Aldrich) to analyze cellulase activity and 0.2 % xylan from beechwood (Sigma-Aldrich) to analyze hemicellulase (xylanase) activity. A drop (15 μL) of bacterial culture grown overnight (100 rpm at 25 °C) was introduced on to agar plate. Fungal strains (agar plugs of 25 mm^2^) were placed in the center of the agar plate. All assays were performed in duplicate using as a negative control MMA without a carbon source. The plates were incubated at 28 °C for 36 h and, after evaluation of growth, they were flooded with Gram iodine [19] for the detection of CMC-ase and xylanase activity. We screened a total of 36 bacterial and 13 fungal strains. CMC and xylan degradation was indicated by detection of clearing zones (haloes) around the colonies. A cut-off value of more than 2.0 mm was considered as a positive result.

## Results

### Bacterial and Fungal Abundances Along the Sequential Batch Transfers

**Fig. 1:**
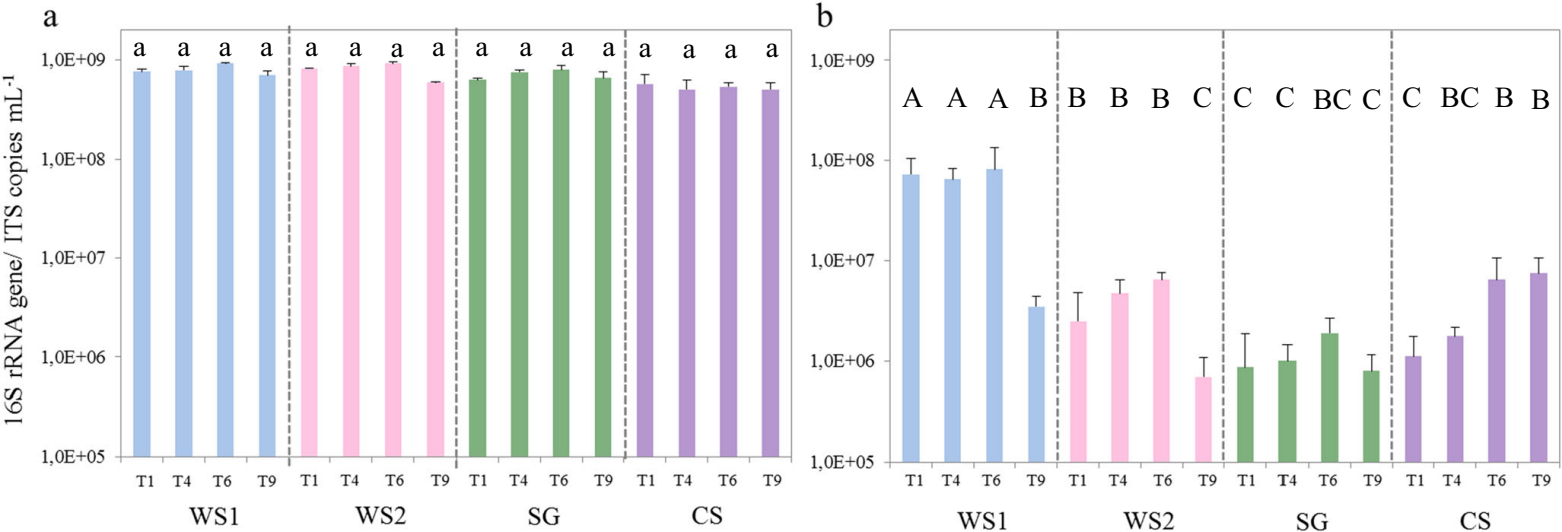
Copy numbers (*y* axis) of (**a**) bacterial 16S rRNA gene and (**b**) fungal ITS region across transfers 1, 4, 6 and 9 for all treatments. Transfers and treatments are indicated on the *x* axis. *Error bars* represent the standard deviation of the means of three independent replicates. *Different lowercase letters (a)* refer to differences among the 16S rRNA gene abundances within treatments and *uppercase letters (A-C)* to differences among ITS1 region abundances across treatments (ANOVA, *p* < 0.05). Abbreviations: *WS1* - wheat straw pH 7.2, *WS2* - wheat straw pH 9.0, *SG* - switchgrass pH 7.2, *CS* - corn stover pH 7.2

In all batches of all treatments, the initial population sizes revolved around ~10^5^ bacterial cells mL^−1^, and these increased to ~10^8^ bacterial cells mL^−1^ during incubation. Invariably, the cell densities increased rapidly to ~10^7^ to ~10^8^ over the first 3 days of incubation, indicating the occurrence of a phase of rapid growth, which was followed by a slower increase to the final cell densities. This pattern was consistently observed across treatments and transfers. No growth was observed in the control treatments (i.e., no substrate with inoculum and no inoculum with substrates). Overall, the qPCR measurements revealed the copy numbers of the bacterial 16S rRNA gene to vary from 5.05 ±1.17 × 10^8^ mL^−1^ (CS) (mean ± SD) to 9.22 ±0.21 × 10^8^ mL^−1^ (WS1) after growth, whereas these were 1000-fold lower at the onset of each growth step. Thus, for all treatments, the bacterial densities reached rather similar maximum levels from similar initial levels (Fig. 1a). In contrast, the abundances of fungal propagules (after growth at each step) showed larger variation across transfers and treatments ranging from 6.94 ±3.84 × 10^5^ (WS2) to 8.18 ±5.30 × 10^7^ ITS1 copies mL^−1^ (WS1) (Fig. 1b). Remarkably, significantly higher numbers of fungal propagules were observed in the WS than in the SG and CS samples (ANOVA, *p* < 0.05).

### Substrate Weight Loss

**Fig. 2:**
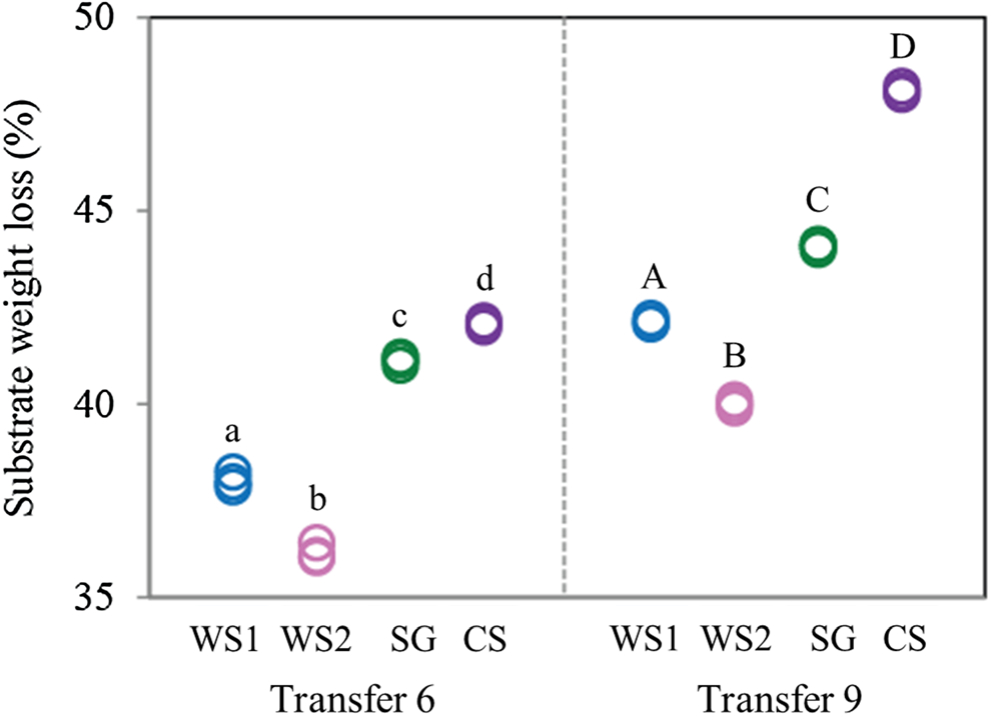
Substrate weight loss (%) of different substrates in the transfers 6 and 9. *Different lowercase letters (a-d)* refer to differences among treatments in T6 and *uppercase ones (A-D)* to differences among treatments, at T9 (ANOVA, *p* < 0.01). Abbreviations: *WS1* - wheat straw pH 7.2, *WS2* - wheat straw pH 9.0, *SG* - switchgrass pH 7.2, *CS* - corn stover pH 7.2

We evaluated substrate weight loss after microbial consortium development on the different plant biomass along transfers 6 (T6) and 9 (T9) through gravimetric determination of dry substrate. Following T6, substrate weight losses were minimally 36.05 ± 0.04 % (WS2) and maximally 42.06 ± 0.06 % (CS). These increased significantly at T9 when values of 42.04 ± 0.06 % (WS2, minimum) and 48.04 ± 0.04 % (CS, maximum) were found. The values were significantly different (ANOVA, *p* < 0.01, Fig. 2).

### Lignocellulosic Composition of WS, CS and SG Substrates and Degradation Rate

**Fig. 3:**
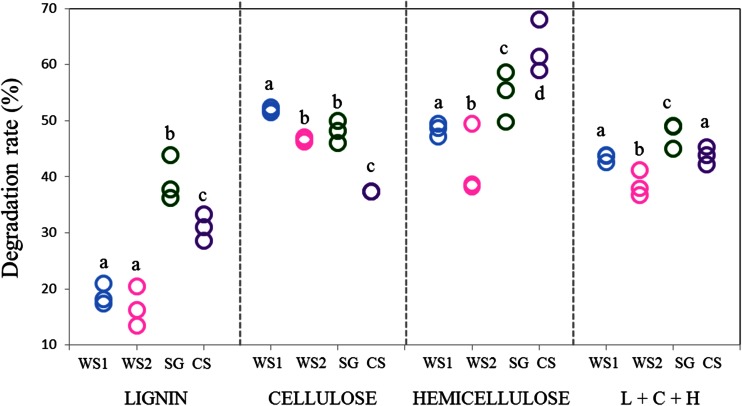
Degradation rates of lignocellulosic components of substrates in transfer 9. *Different letters (a-d)* refer to differences among the means of treatments (ANOVA, *p* < 0.01). Abbreviations: *L* - lignin, *C* - cellulose, *H* - hemicellulose, *WS1* - wheat straw pH 7.2, *WS2* - wheat straw pH 9.0, *SG* - switchgrass pH 7.2, *CS* - corn stover pH 7.2

The composition of all plant matter in terms of lignin, cellulose and hemicellulose (xylan) was measured for all substrates. Moreover, we measured these parameters before and after consortial growth in transfer 9, allowing calculation of the degradation rate of these components (Table 1; Fig. 3). WS, CS and SG differed within limits with respect to the presence of the main measured components lignin, cellulose, and hemicellulose (Table 1). In terms of degradation by the T9 consortium, we found the highest lignin degradation rate in treatment SG (39.32 ± 4.04 %), whereas the highest degradation rate of cellulose occurred in treatment WS1 (51.92 ± 0.41 %) and of hemicellulose in CS (62.79 ± 4.69 %). Moreover, considering the total degradation of lignocellulosic components (i.e., lignin + cellulose + xylan), SG turned out to be the most efficiently degraded substrate (47.67 ± 2.33 %), followed by CS (43.81 ± 1.53 %), WS1 (43.40 ± 0.69 %) and WS2 (38.60 ± 2.29 %).

**Table 1:** Lignocellulosic composition (lignin, cellulose, and hemicellulose) of substrates*

Substrate	Lignin (%)	Cellulose (%)	Hemicellulose (%)
WS^a^	22.2 ± 0.8	45.5 ± 1.3	31.3 ± 0.9
WS1^b^	18.0 ± 1.0	21.9 ± 0.6	16.1 ± 0.7
WS2^b^	18.4 ± 0.5	24.2 ± 0.8	18.1 ± 1.6
SG^a^	22.3 ± 0.9	45.9 ± 1.5	24.0 ± 1.0
SG^b^	13.5 ± 1.0	23.8 ± 1.0	10.9 ± 1.1
CS^a^	25.2 ± 0.8	40.3 ± 1.7	30.3 ± 0.2
CS^b^	17.4 ± 0.6	25.2 ± 0.3	11.2 ± 1.0

Abbreviations: *WS1* - wheat straw pH 7.2, *WS2* - wheat straw pH 9.0, *SG* - switchgrass pH 7.2, *CS* - corn stover pH 7.2

^a^Substrate before incubation

^b^Substrate after incubation

*Average and standard deviation of three replicates

### Analysis of Microbial Consortium Structures by PCR-DGGE

**Fig. 4:**
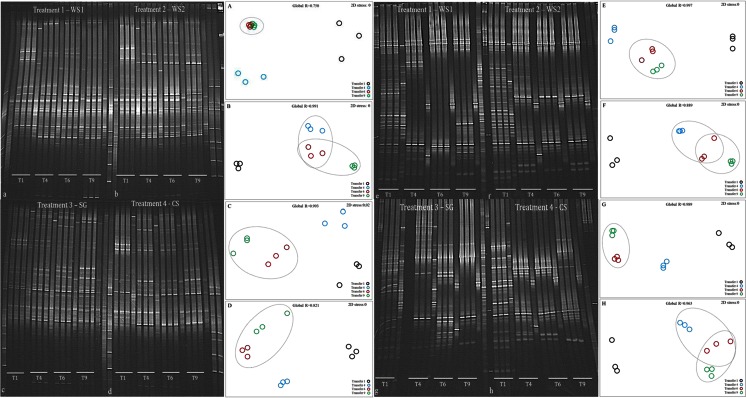
Community fingerprints (PCR-DGGE) of bacterial and fungal communities along transfers 1, 4, 6 and 9 on different substrates; (*a*) WS1, (*b*) WS2, (*c*) SG and (*d*) CS, for bacterial communities and (*e*) WS1, (*f*) WS2, (*g*) SG and (*h*) CS, for fungal communities. *A*, *B*, *C* and *D* represent nMDS and statistical analyses (ANOSIM; global *R* value) for bacterial communities in the different substrates and **E**, **F**, **G** and **H** for fungal communities in the different substrates. Abbreviations: *WS1* - wheat straw pH 7.2, *WS2* - wheat straw pH 9.0, *SG* - switchgrass pH 7.2, *CS* - corn stover pH 7.2

Using total consortium DNA, bacterial 16S rRNA gene and ITS region-based PCR-DGGE analyses were used to evaluate the evolution of the community structures across the sequential batches per treatment (Figs. 3 and 4). The source inoculum contained a “cloud” of bands, estimated to encompass at least 60 bands in the bacterial fingerprints and >45 bands in the fungal ones. The data further showed that the triplicates of each treatment in each transfer consistently depicted similar communities per treatment, with reduced richness as compared to the source inoculum. This was true for both the bacterial and fungal communities.

For treatments WS1, WS2 and CS, the bacterial community fingerprints showed highest numbers of bands (here taken as proxies for the richness of dominant organisms) in the initial transfer, with decreases afterwards (from initially 13, 13 and 11 to finally 10, 11 and 8 bands, respectively) (Fig. 4 (a, b, d)). On the other hand, band numbers increased in the SG consortia from initially 8 to finally 13 bands along the transfers (Fig. 4 (c)). Stability in the community compositions was observed after transfer 6 in WS1, SG and CS and after transfer 4 in WS2 (Fig. 4 (A–D)).

Fungal richness revealed a trend that was similar to that observed for bacterial richness along the transfers. In transfer 1, WS1, WS2, SG and CS showed 23, 23, 10 and 15 bands respectively, which declined to respectively 7, 6, 5 and 7 bands in transfer 9. This trend was consistent across the replicates (Fig. 4 (e–h)). The fungal community structures reached stability after transfer 6 in WS1 and SG and after transfer 4 for WS2 and CS (Fig. 4 (E–H)).

**Fig. 5:**
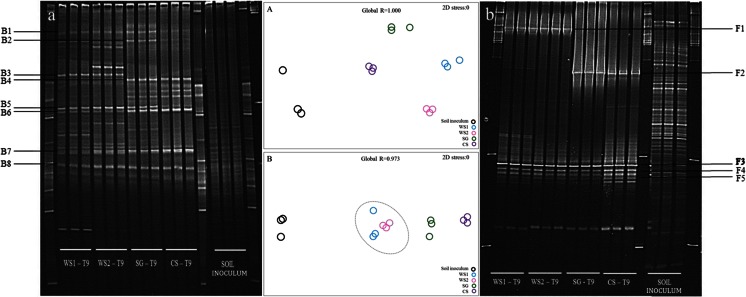
Community fingerprinting (PCR-DGGE) for (*a*) bacterial and (*b*) fungal communities in the final consortia on different substrates and for the original soil inoculum. *A* and *B* represent nMDS and statistical analyses (ANOSIM; global *R* value) for bacterial and fungal communities respectively. To details about B1 - B8 and F1 - F5, see text. Abbreviations: *WS1* - wheat straw pH 7.2, *WS2* - wheat straw pH 9.0, *SG* - switchgrass pH 7.2, *CS* - corn stover pH 7.2 *T9* - transfer 9

The T9 PCR-DGGE profiles were then compared across the treatments (Fig. 5). Cluster analysis of these profiles revealed ~40 and ~60 % of differences across treatments for the bacterial and fungal consortia, respectively (Supplementary Fig. S1). Clearly, substrate type, next to pH for the treatments using WS, drove the bacterial community structures (Fig. 5 (A)), these being partially variable and partially stable. Thus, a common core, consisting of five bands, was observed across all treatments (i.e., B1, B5, B6, B7 and B8; Fig. 4 (a)). Next to this core, another band was found to be common between treatments WS1 and WS2 (B3; Fig. 5 (a)) and yet another one between treatments SG and CS (B4; Fig. 5 (a)). Using co-migration analyses, we found that the core consortium bands B1, B5, B6, B7 and B8 were similar to those from the strains (see later) affiliated with *Sphingobacterium kitahiroshimense*, *Enterobacter amnigenus*, *Raoultella terrigena*, *Pseudomonas putida* and *Stenotrophomonas rhizophila*, respectively (Fig. 5 (a)). Band B2 was assigned to *Paenibacillus xylanexedens*, which was only present in the SG consortium.

With respect to the fungal communities, substrate type also was a main factor driving the community structures. Treatments WS1 and WS2, which used the same substrate (wheat straw) under different pH values, incited similar fungal community structures (Fig. 5 (B)). Three common bands, potentially reflecting the existence of a fungal core community, were observed in the final consortia across all treatments (F3, F4 and F5; Fig. 5 (b)) next to a common one for treatments WS1 and WS2 (F1; Fig. 5 (b)) and another one for treatments SG and CS (F2; Fig. 5 (b)).

### Isolation of Bacterial and Fungal Strains from Enriched Cultures

Totals of 36 bacterial and 13 fungal strains recovered from each treatment (Supplementary Fig. S2a, S2b) at T9 were presumptively identified by 16S rRNA gene (bacteria) and ITS1 (fungi) sequencing (Table 2). Specifically, 11, 8, 9 and 8 bacterial and 4, 3, 3 and 3 fungal isolates recovered from WS1, WS2, SG and CS respectively, were thus identified.

**Table 2:** Taxonomic affiliation and enzymatic activity of bacterial and fungal isolates from transfers 9 of all treatments

T	Isol	Identification	Cov	Ident	Access no.	Enz act
Bacteria						
WS1	1	*Raoultella terrigena* Rsh21 16S ribosomal RNA gene	100 %	100 %	KF796627.1	x
	2	*Sphingobacterium mizutani* Ht8-22 16S ribosomal RNA gene	99 %	99 %	JF899285.1	–
	3	*Raoultella terrigena* PSB15 16S ribosomal RNA gene	100 %	99 %	HQ242728.1	x
	4	*Klebsiella terrigena* 16S ribossonal RNA gene SW4 partial	100 %	99 %	Y17670.1	x
	5	*Sphingobacterium mizutani* Ht8-22 16S ribosomal RNA gene	100 %	99 %	JF899285.1	–
	6	*Sphingobacterium mizutani* Ht8-22 16S ribosomal RNA gene	99 %	99 %	JF899285.1	–
	7	*Pseudomonas putida* ATCC 17494 16S ribosomal RNA gene	100 %	100 %	AF094740.2	–
	8	*Pseudomonas putida* 214-D 16S ribosomal RNA gene, partial sequence	100 %	100 %	EF615008.1	–
	9	*Stenotrophomonas rhizophila* BG9 16S ribosomal RNA gene	100 %	100 %	KJ997741.1	x
	10	*Raoultella terrigena* PSB15 16S ribosomal RNA gene	100 %	99 %	HQ242728.1	x
	11	*Enterobacter amnigenus* h-14 16S ribosomal RNA gene	100 %	100 %	KC139434.1	–
WS2	12	*Pseudomonas putida* 214-D 16S ribosomal RNA gene, partial sequence	100 %	100 %	EF615008.1	–
	13	*Raoultella terrigena* PSB15 16S ribosomal RNA gene	100 %	100 %	HQ242728.1	x
	14	*Sphingobacterium kitahiroshimense* 10C 16S ribosomal RNA gene	100 %	99 %	NR_041636.1	x
	15	*Pseudomonas vranovensis* IBFC2012-27 16S ribosomal RNA gene	100 %	99 %	KC246044.1	x
	16	*Stenotrophomonas rhizophila* BG9 16S ribosomal RNA gene	100 %	100 %	KJ997741.1	x
	17	*Raoultella terrigena* RN16 16S ribosomal RNA gene	100 %	100 %	KC790281.1	–
	18	*Sphingobacterium kitahiroshimense* 10C 16S ribosomal RNA gene	100 %	99 %	NR_041636.1	x
	19	*Stenotrophomonas rhizophila* BG9 16S ribosomal RNA gene	100 %	100 %	KJ997741.1	x
SG	20	*Sphingobacterium kitahiroshimense* 10C 16S ribosomal RNA gene	100 %	99 %	NR_041636.1	x
	21	*Sphingobacterium kitahiroshimense* 10C 16S ribosomal RNA gene	100 %	99 %	NR_041636.1	x
	22	*Raoultella terrigena* Rsh21 16S ribosomal RNA gene	100 %	99 %	KF796627.1	x
	23	*Raoultella terrigena* Rsh21 16S ribosomal RNA gene	100 %	99 %	KF796627.1	x
	24	*Enterobacter amnigenus* h-14 16S ribosomal RNA gene	100 %	100 %	KC139434.1	–
	25	*Enterobacter amnigenus* h-14 16S ribosomal RNA gene	100 %	100 %	KC139434.1	–
	26	*Paenibacillus xylanexedens* JDG191 16S ribosomal RNA gene	82 %	99 %	JX035957.1	x
	27	*Delftia tsuruhatensis* LAM 29 16S ribosomal RNA gene	100 %	99 %	EU019989.1	–
	28	*Enterobacter amnigenus* h-14 16S ribosomal RNA gene	100 %	100 %	KC139434.1	–
CS	29	*Sanguibacter inulinus* 16S ribosomal RNA: ST50 clone: NTS14	99 %	100 %	AB920571.1	x
	30	*Sphingobacterium faecium* Gen5 16S ribosomal RNA gene	100 %	99 %	KJ726588.1	–
	31	*Stenotrophomonas rhizophila* BG9 16S ribosomal RNA gene	100 %	100 %	KJ997741.1	x
	32	*Sphingobacterium anhuiense* CW 186 16S ribosomal RNA gene	100 %	99 %	NR_044477.1	–
	33	*Sanguibacter inulinus* 16S ribosomal RNA: ST50 clone: NTS14	100 %	100 %	AB920571.1	x
	34	*Pseudomonas alkylphenolia* KL28, complete genome	100 %	99 %	CP009048.1	–
	35	*Stenotrophomonas rhizophila* BG9 16S ribosomal RNA gene	100 %	100 %	KJ997741.1	x
	36	*Comamonas jiangduensis* Amp3 16S ribosomal RNA gene	100 %	99 %	KJ726553.1	x
Fungi						
WS1	1	*Coniochaeta ligniaria* 2w1F 18S ribosomal RNA gene	93 %	99 %	KF285992.1	x
	2	*Coniochaeta ligniaria* 2w1F 18S ribosomal RNA gene	91 %	99 %	KF285992.1	x
	3	*Coniochaeta ligniaria* 2w1F 18S ribosomal RNA gene	91 %	99 %	KF285992.1	x
	4	*Acremonium* sp. 11665 DLW-2010 18S ribosomal RNA gene	98 %	96 %	GQ867783.1	x
WS2	5	*Coniochaeta ligniaria* 2w1F 18S ribosomal RNA gene	92 %	99 %	KF285992.1	x
	6	*Acremonium* sp. 11665 DLW-2010 18S ribosomal RNA gene	98 %	96 %	GQ867783.1	x
	7	*Acremonium* sp. 11665 DLW-2010 18S ribosomal RNA gene	99 %	97 %	GQ867783.1	x
SG	8	*Coniochaeta ligniaria* 2 t2.1 F 18S ribosomal RNA gene	94 %	96 %	KF285995.1	x
	9	*Coniochaeta ligniaria* 2w1F 18S ribosomal RNA gene	95 %	99 %	KF285992.1	x
	10	*Acremonium* sp. 11665 DLW-2010 18S ribosomal RNA gene	98 %	95 %	GQ867783.1	x
CS	11	*Coniochaeta ligniaria* 2w1F 18S ribosomal RNA gene	94 %	97 %	KF285992.1	x
	12	*Acremonium* sp. 11665 DLW-2010 18S ribosomal RNA gene	95 %	96 %	GQ867783.1	x
	13	*Acremonium* sp. 11665 DLW-2010 18S ribosomal RNA gene	98 %	97 %	GQ867783.1	x

Abbreviations: *WS1* - wheat straw pH 7.2, *WS2* - wheat straw pH 9.0, *SG* - switchgrass pH 7.2, *CS* - corn stover pH 7.2, *T* - treatment, *Isol* - isolate, *Cov* - coverage, *Ident* - identity, *Access No.* - access number, *Enz.Act* - enzymatic activity

**Fig. 6:**
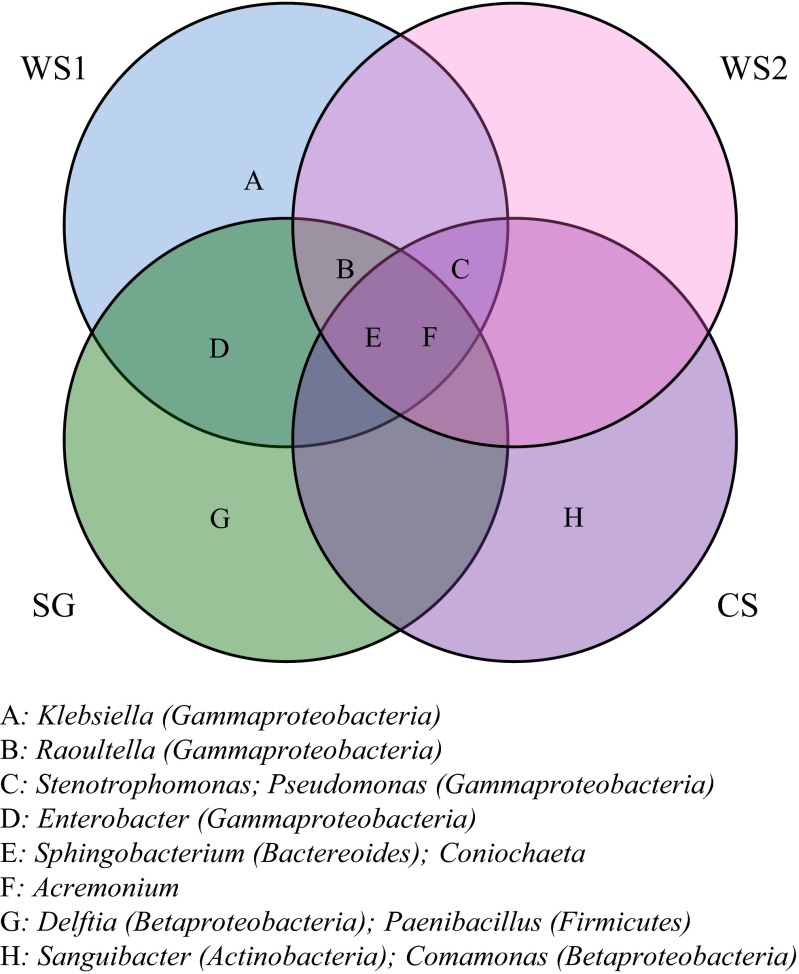
Venn diagram indicating unique and common bacterial and fungal strains across all treatments. Abbreviations: *WS1* - wheat straw pH 7.2, *WS2* - wheat straw pH 9.0, *SG* - switchgrass pH 7.2, *CS* - corn stover pH 7.2

The bacterial strains obtained from WS1 were affiliated (>99 % identity with NCBI database entries; number of strains indicated between parentheses) with *R. terrigena* (3), *S. kitahiroshimense* (3), *K. terrigena* (1), *P. putida* (2), *S. rhizophila* (1), and *E. amnigenus* (1). Strains from WS2 were affiliated with *Pseudomonas putida* (2), *R. terrigena* (2), *S. kitahiroshimense* (2), and *S. rhizophila* (2). Treatment SG yielded strains affiliated with *S. kitahiroshimense* (2), *R. terrigena* (2), *E. amnigenus* (3), *P. xylanexedens* (1), and *D. tsuruhatensis* (1). Strains obtained from treatment CS were affiliated with *S. rhizophila* (2), *S. kitahiroshimense* (2), *P. putida* (1), *C. jiangduensis* (1), and *S. inulinus* (2). The isolated fungal strains for all treatments were affiliated (>95 % identity with NCBI database entries) with *C. ligniaria* and *Acremonium* sp*.* (Table 2; Fig. 6).

### Bacterial and Fungal (Hemi)cellulolytic Activities

In the light of their presumed dominance in the PCR-DGGE profiles, we tested the microorganisms affiliated with *S. kitahiroshimense*, *E. amnigenus*, *R. terrigena*, *P. putida* and *S. rhizophila*, next to *P. xylanexedens*, for their ability to deconstruct plant biomass. We thus tested (hemi)cellulolytic activity for these, next to other isolates (CMC-ase and xylanase). Twenty one bacterial strains derived from treatments WS1 (5/11), WS2 (6/8), SG (5/9), and CS (5/8), respectively, showed positive CMC-ase as well as xylanase activities. Indeed, the strains affiliated with *Sphingobacterium kitahiroshimense*, *R. terrigena*, *P. vranovensis*, *S. rhizophila* (bacteria), *C. lignaria*, and *Acremonium* sp. (fungi), presumably belonging to the microbial “cores”, showed positive (hemi)cellulolytic activity. In addition, specialist isolates (*P. xylanexedens*, *S. inulus*, and *C. jiangduensis*) also showed CMC-ase and xylanase activity (Table 2 and Supplementary Fig. S2c; S2d).

## Discussion

The development of efficient microbial consortia to deconstruct plant biomass is of great industrial interest. The biodegradation process involves a network of enzymatic transformations that requires timely production by microbial cells and extracellular availability. Moreover, stress conditions might be better endured by consortia than by single strains as a result of community interactions. In this study, different plant biomass sources were used to produce specific microbial consortia for lignocellulose degradation. The dilution-to-stimulation approach used worked well, as verified by observing the growth of bacterial cells in each step, which reached up to ~10^8^ cells mL^-1^ after 5 to 6 days of incubation. Previous work from our lab [18]—using a similar approach to enrich lignocellulose degraders—observed that maximal cell densities of 10^7^–10^8^ cells mL^-1^ were reached after 6 to 8 days. However, lower temperatures and shaking conditions were used than the ones used in this study (i.e., 25 °C and 100 rpm). Consistent with Jimenez et al. [18], the fungal communities did not build up high densities in the enrichment systems (Fig. 1b), with ITS1 gene copy numbers remaining well below the bacterial 16S rRNA gene copy numbers.

On all substrates, the microbial consortia, across all treatments, effected enhanced substrate weight loss from transfers 6 to 9, with values ranging from a minimum of about 36 % (WS2, in transfer 6) up to around 48 % (CS, in transfer 9). In addition, treatments SG and CS had higher values of substrate weight loss than treatments WS1 and WS2 (Fig. 2). Such weight loss data were roughly consistent with the overall FTIR-based data (Table 1). Thus, the plant biomass degradative microbial consortia were apparently “trained” to become more efficient in the degradation process over time. Xu et al. [41], testing the weight loss of corn stover in a culture of white rot fungus *Irpex lacteus*, described a substrate weight loss of ~20 % after 40 days of incubation. Similarly, Baldrian et al. [4] showed the weight loss of wheat straw during growth of *Pleurotus ostreatus* to be at the level of ~30 % after 20 days of incubation. Thus, in spite of the fact that we do not provide a side-by-side comparison, we conclude that mixed microbial consortia (i.e., consisting of both bacterial and fungal partners) have potentially higher biodegradative performance than single-isolate cultures.

The FTIR-based analyses showed treatment SG to have the highest lignin degradation rate (ca. 39 %), while the highest rates for cellulose and hemicellulose were obtained in the WS1 (ca. 52 %) and CS (ca. 63 %) treatments, respectively (Fig. 3). Whereas several previous studies have addressed plant biomass degradation by breeding different microbial consortia [5, 17, 18, 44], none has studied the influence of different lignocellulose substrates or different pH conditions as factors driving the enrichment of specific microbial consortia once the same microbial source is used as an inoculum. Here, we clearly show that substrate type, next to pH (for treatments using WS), are major driver of the microbial consortia that are bred from one source inoculum. Such consortia were consistent across replicates yet were found to be composed of different members across treatments (Fig. 5 (a)). Given the fact that the lignocellulose compositions of the three used substrates were roughly similar (Table 1) and taking on board the evidence that the rates of decomposition of these different compounds were different across the treatments (Table 1), we can discern a scientific basis for the divergent microbial consortia emerging in the different substrates. These lie either or both in the presumed differences in soluble carbohydrate and sugar compositions or in the intricate bonds and/or branching within and between the three substrates that make up the lignocellulose moieties of the three plants. However, with respect to the bacterial parts of the consortia, we detected a restricted “core” consortium across the treatments, next to a treatment-specific one. The apparent “core” was consistently composed of organisms affiliated with *Sphingobacterium kitahiroshimense*, *Raoultella terrigena*, *Pseudomonas putida* and *Stenotrophomonas rhizophila*. Interestingly, these genera were also found to become abundant in previously-bred microbial consortia using (un)treated wheat straw as the carbon source [18]. Presumably, these consist of “generalists” that grow upon common target in the diverse plant biomasses.

Moreover, the fungal consortia also revealed stable structures that were different from each other across the treatments. On top of that, treatments WS1 and WS2 revealed the emergence of statistically similar fungal community structures (Fig. 5 (b)), indicating a general lack of effect of pH conditions on these communities, in this case specifically for the WS treatment. We cannot easily explain this fact, as Jiménez et al. [18] noticed that fungal community structures enriched with wheat straw and torrified wheat straw were very dissimilar from each other.

Furthermore, a suite of highly active isolates that likely represent members of the core microbiota was obtained, and their analysis yielded important observations. First, as activity detection included the observation of haloes, the produced enzymes were externally secreted by the cells. Secretion is a critical, yet overlooked, bottleneck in studies that aim at the establishment of efficient microbial degrader consortia. Our *Raoultella and Klebsiella* isolates (*Enterobacteriales*) showed extracellular (xylanolytic/cellulolytic) activities, suggesting that these bacteria have metabolic roles in plant polymer degradation. This finding is possibly congruent with studies that showed members of the *Enterobacteriales* abound in insect herbivore microbiomes [3, 35].

The finding of *Stenotrophomonas*—like organisms (part of the core consortia) showing (hemi)cellulolytic activity—corroborates data obtained by Qi et al. [32]. The latter study on lignocellulosic substrate bioconversion by yellow mealworm gut microbiomes produced a degrading microbial consortium that contained key *Stenotrophomonas* strains for the degradation of lignocellulosic material.

Interestingly, bacterial strains retrieved from CS (affiliated with *Comamonas* and *Sanguibacter*) and SG (affiliated with *Paenibacillus*) also showed degrader activities, suggesting these are potentially active lignocellulose degraders. Wang et al. [45] reported organisms affiliated with *Paenibacillus* to be key degraders of lignocellulosic substrates (from reeds), whereas Cook et al. [7] found *Sanguibacter suarezii* to degrade CMC, starch, methylumbelliferyl (MUF)-xylopyranoside, MUF-arabinofuranoside and MUF-glucopyranoside. Finally, it is noteworthy that *Coniochaeta* - and *Acremonium*- like fungi with CMC-ase and xylanase activities, were consistently found across all treatments. Thus, such organisms might have key roles in the core degradative consortium. The genus *Coniochaeta* encompasses filamentous fungi that are active in the degradation of decaying wood in soil and are probably involved in hemicellulose degradation [26]. Recently, *Plectosphaerella* (which is highly related to *Acremonium*) has been reported to utilize xylose and CMC, yielding lipids [24, 36]. Thus, the production of lipids by such organisms—using lignocellulose as a substrate—may constitute a metabolic pathway to be explored in order to yield oil-rich compounds—a process with high economic competitiveness [25].

In summary, we developed four lignocellulose-degrading microbial consortia from forest soil using three different plant substrates. Substrate type was found to be the major driver of the composition of the bacterial and fungal communities in the final consortia, as evidenced by PCR-DGGE community profiling along the enrichments. Moreover, a common core consortium of low richness was detected. Further understanding of the biotic interactions in the bred consortia will pave the way for the establishment of an efficient multispecies-based process for lignocellulose degradation.

## Electronic supplementary material

Supplementary Figure S1 Cluster analysis of DGGE profiles from transfer 9. for all treatments and soil. targeting (a) Bacterial 16S rRNA gene and (b) Fungal ITS region. (PPTX 198 kb)

Supplementary Figure S2 (a) Isolation and (b) purification of bacterial and fungal isolates from the transfer 9 for all treatments and halo formation in (c) bacterial and (d) fungal isolates in the enzymatic test. (PPTX 992 kb)

ESM 1 (DOCX 15 kb)
