# Supplementary figures and images for: Soil-Derived Microbial Consortia Enriched with Different Plant Biomass Reveal Distinct Players Acting in Lignocellulose Degradation
Source: Microb Ecol. 2015 Oct 20;71:616–27. doi: 10.1007/s00248-015-0683-7 (PMC4788684; doi:10.1007/s00248-015-0683-7)

## Slide 1
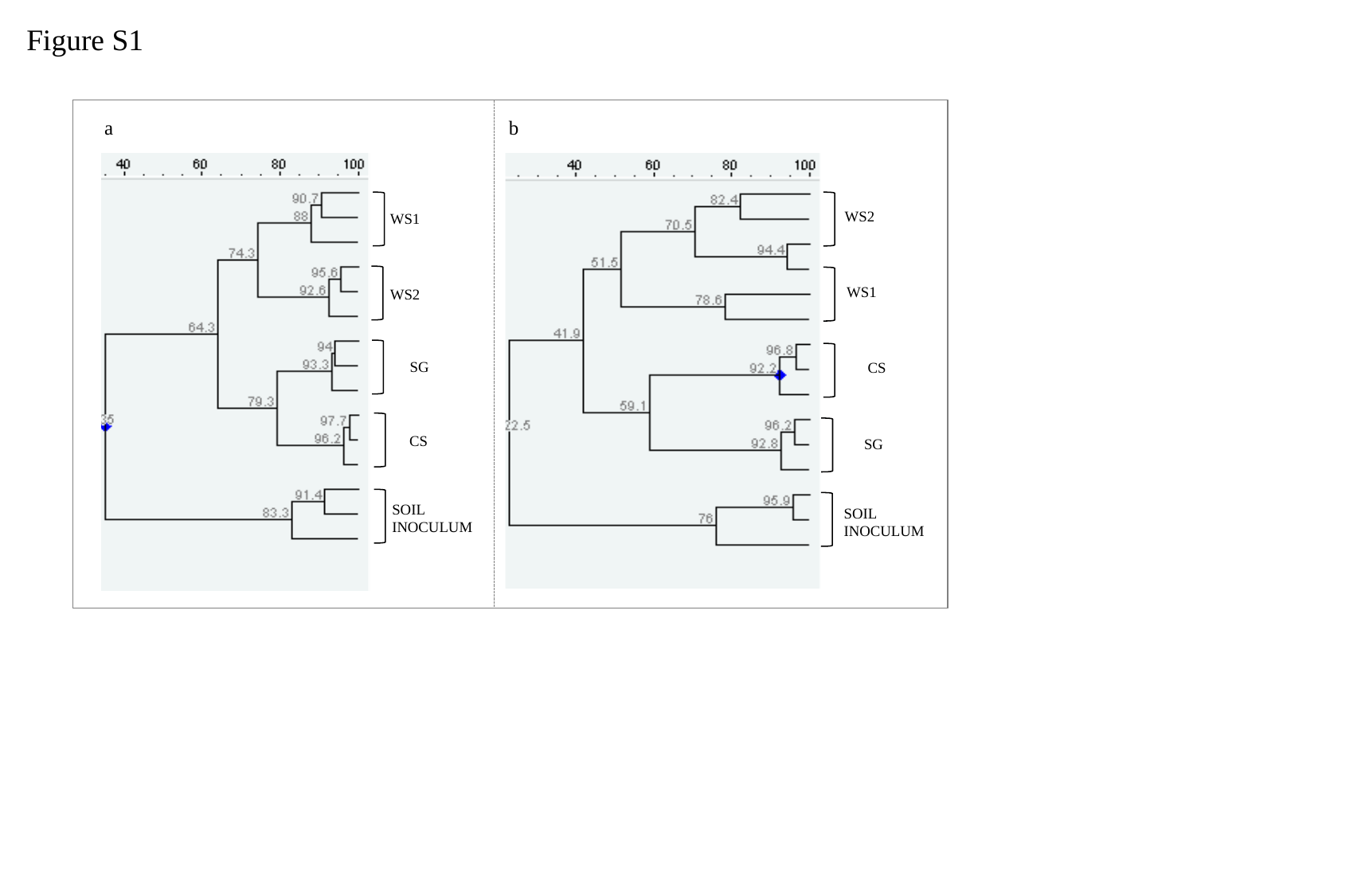

Figure S1
b
WS2
WS1
WS1
WS2
SG
CS
CS
SG
SOIL
INOCULUM
SOIL
INOCULUM
a

Supplement: Supplementary file 1 — Cluster analysis of DGGE profiles from transfer 9. for all treatments and soil. targeting (a) Bacterial 16S rRNA gene and (b) Fungal ITS region. (PPTX 198 kb) [file 248_2015_683_MOESM1_ESM.pptx]

## Slide 1
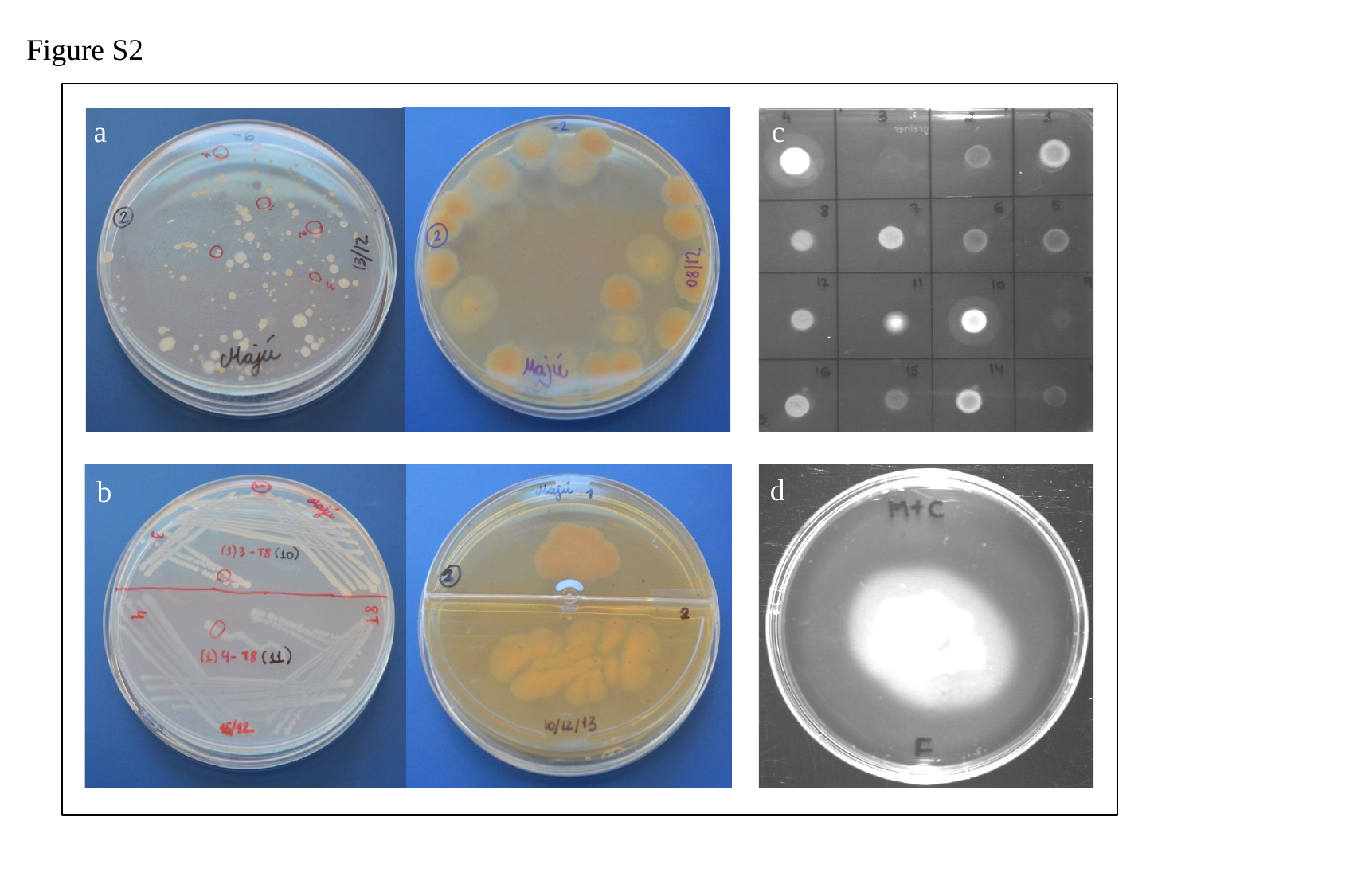

Figure S2
c
c
a
b
d
d

Supplement: Supplementary file 2 — (a) Isolation and (b) purification of bacterial and fungal isolates from the transfer 9 for all treatments and halo formation in (c) bacterial and (d) fungal isolates in the enzymatic test. (PPTX 992 kb) [file 248_2015_683_MOESM2_ESM.pptx]
