# Supplementary material for: Soil-Derived Microbial Consortia Enriched with Different Plant Biomass Reveal Distinct Players Acting in Lignocellulose Degradation
Source: Microb Ecol. 2015 Oct 20;71:616–27. doi: 10.1007/s00248-015-0683-7 (PMC4788684; doi:10.1007/s00248-015-0683-7)
Supplement: Supplementary file 3 — (DOCX 15 kb) [file 248_2015_683_MOESM3_ESM.docx]

**Supplementary information and supplementary information legends**

**Supplementary Table S1.** Cellulose. hemicellulose and lignin mixtures used to obtain reference spectra. Values are expressed in %.

| Mixtures | Lignin (%) | Cellulose (%) | Hemicellulose (%) |
| --- | --- | --- | --- |
| 1 | 100^a^ | 0 | 0 |
| 2 | 50 | 25 | 25 |
| 3 | 25 | 50 | 25 |
| 4 | 25 | 25 | 50 |
| 5 | 75 | 25 | 0 |
| 6 | 25 | 75 | 0 |
| 7 | 0 | 25 | 75 |
| 8 | 0 | 75 | 75 |

**Supplementary Figure S1.** Cluster analysis of DGGE profiles from transfer 9. for all treatments and soil. targeting (a) Bacterial 16S rRNA gene and (b) Fungal ITS region.

**Supplementary Figure S2.** (a) Isolation and (b) purification of bacterial and fungal isolates from the transfer 9 for all treatments and halo formation in (c) bacterial and (d) fungal isolates in the enzymatic test.
